# Application of YOLOv4 Algorithm for Foreign Object Detection on a Belt Conveyor in a Low-Illumination Environment

**DOI:** 10.3390/s22186851

**Published:** 2022-09-10

**Authors:** Yiming Chen, Xu Sun, Liang Xu, Sencai Ma, Jun Li, Yusong Pang, Gang Cheng

**Affiliations:** 1 Shandong Zhongheng Optoelectronic Technology Co., Ltd., Zaozhuang 277000, China; 2 School of Mechatronic Engineering, China University of Mining and Technology, Xuzhou 221116, China; 3 Faculty Mechanical, Maritime and Materials Engineering, Delft University of Technology, 2628 Delft, The Netherlands

**Keywords:** belt conveyor, machine vision, KinD++ algorithm, YOLOv4 algorithm, low-light enhancement

## Abstract

The most common failures of belt conveyors are runout, coal piles and longitudinal tears. The detection methods for longitudinal tearing are currently not particularly effective. A key study area for minimizing longitudinal belt tears with the advancement of machine learning is how to use machine vision technology to detect foreign items on the belt. In this study, the real-time detection of foreign items on belt conveyors is accomplished using a machine vision method. Firstly, the KinD++ low-light image enhancement algorithm is used to improve the quality of the captured low-quality images through feature processing. Then, the GridMask method partially masks the foreign objects in the training images, thus extending the data set. Finally, the YOLOv4 algorithm with optimized anchor boxes is combined to achieve efficient detection of foreign objects in belt conveyors, and the method is verified as effective.

## 1. Introduction

With the belt conveyor being widely used in China’s coal production process in recent years, effectively ensuring the regular operation of the belt conveyor has become a key concern for coal companies. Effective belt tearing reduction has become a crucial component of regular belt conveyor operation since the belt is the component most crucial and susceptible to damage in a belt conveyor. The majority of research on belt conveyor tear protection focuses on the belt’s ability to detect tears. Detecting whether the belt is torn can stop the belt in time at the early stage of belt tearing and reduce belt damage [1,2]. However, the belt conveyor foreign body detection starts from the cause of belt tearing, which can achieve belt protection from the root cause.

The two conventional techniques for detecting conveyor foreign objects are the ray method and the infrared detection method. The ray method requires measuring the release of each coal release bracket, and the sensor itself is more expensive, which drives up the cost of mining. Hence, the application is narrow [3,4,5,6]. The infrared detection method can take a variety of roof plates as working objects and is responsive. However, this method’s recognition accuracy is influenced by the temperature of the coal mining machine and the water spray process used to remove dust [7,8,9]. With the development of technology, methods based on image recognition technology to achieve belt conveyor foreign object detection are beginning to be applied. Zhao et al. studied a coal gangue image processing and recognition system based on Da Vinci technology [10]. Li et al. studied a coal gangue recognition method based on image processing [11]. Yu et al. constructed a new coal and gangue image recognition method based on a nonlinear greyscale compression–expansion symbiotic matrix [12].

However, the working environment of coal mine belt conveyors is harsh, with high dust and dim lighting. The quality of the captured images is poor and the brightness is very low. The dataset composed of such images is not conducive to subsequent training, so these low-light images need to be enhanced to highlight the features of their objects. Retinex theory [13] assumes that color images can be decomposed into two components: reflectance and illumination. The single-scale Retinex algorithm [14] (SSR) and the multi-scale Retinex algorithm [15] (MSR) proposed by Jobson et al. are limited to how the final result is produced, with the output often looking unnatural and over-enhanced in places.

Meanwhile, in the process of foreign object detection in belt conveyors, in addition to pre-processing the acquired field images, classification of the images is also required. With the development of deep learning, various advanced target detection methods have emerged. Ross Girshick proposed R-CNN as the pioneer of target detection using deep learning [16], and later proposed Fast R-CNN for improving these problems in order to solve the problem of too-slow speed during model training and testing [17]. However, Fast R-CNN still failed to achieve end-to-end target detection despite significantly improving speed and accuracy. On the other hand, the YOLO family of algorithms proposed by Joseph Redmon belongs to the real sense of real-time target detection [18], which differs from the two-step target detection algorithm of the R-CNN family. YOLO is based on a separate end-to-end network that solves object detection as a regression problem, completing from the input of the original image to the output of object location and category.

Based on the above analysis, this paper proposes a deep KinD++ [19] algorithm to achieve low-light image enhancement in the harsh environment of mines. It uses YOLOv4 to obtain optimal anchor box width and height values in order to detect foreign objects in belt conveyors efficiently.

## 2. Principle of KinD++ Based Low-Light Image Enhancement Algorithm

**Figure 1:**
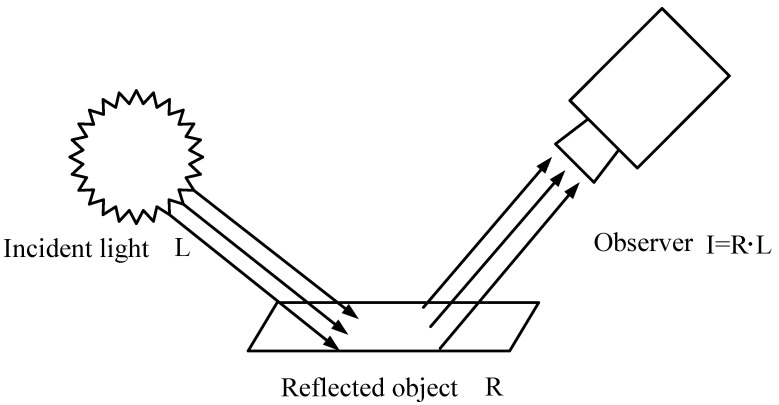
Retinex theoretical object imaging model.

The Retinex theory states that the colors observed by the human eye are not affected by the intensity of external light. This means that the external color observed by the human eye is always the same, regardless of whether it is in a well-lit or poorly lit environment. The color an object exhibits therefore reflects the true properties of the object, which is formed by reflecting light. The ability of an object to reflect light therefore contains the essential properties of the object. Figure 1 shows the schematic diagram of the Retinex theoretical object imaging model.

Retinex theory is based on the idea that the image received by the human eye is the result of the superposition of external light and the reflection of the object. The amount of light intensity causes the image to vary in light and darkness, but the nature of the object’s reflection remains the same. Therefore, to obtain a true picture of a low-light image, the low-light component of the image needs to be removed and the reflective nature of the object in the image needs to be estimated. This allows the image to be restored to its normal illuminated state based on this essential property of the object.

KinD (Kindling the Darkness) is a low-light image enhancement network based on Retinex theory [20], which was proposed by Dr. Y. H. Zhang in 2019. The KinD++ network can be functionally divided into three modules: layer decomposition, reflectance recovery and illumination adjustment.

### 2.1. Layer Decomposition Network

For images, there is no such thing as optimal lighting conditions, and there is no image reflectance map or light map that can be used as a standard reference.

The problem of layer decomposition is essentially undetermined, so additional regularization is important. Using $I_{h}$ and $I_{l}$ to represent the high- and low-light original images of the same scene, respectively, the image pair is fed into the layer decomposition network as input data and decomposed into a reflectance map and a light map at the same time. $R_{h}$ and $R_{l}$ are used to represent the reflection map of the original high-light image and the reflection map of the original low-light image, respectively, and $L_{h}$ and $R_{l}$ are used to represent the illumination map of the original high-light image and the illumination map of the original low-light image, respectively. From Retinex theory, it is known that $R_{h}$ and $R_{l}$ are similar, so first construct the reflectance similarity loss function as follows:

(1) $$L_{rs}^{D}=\left\|R_{l}-{\left.R_{h}\right\|}_{1}\right.$$

where ${\left\|\cdot \right\|}_{1}$ denotes the *L*1 parametrization. The illumination maps $L_{h}$ and $L_{l}$ are known to be segmentally smoothed, so the illumination smoothing loss function is constructed as:

(2) $$L_{is}^{D}={\left\|\left.\frac{\nabla L_{l}}{\max(\left|\nabla I_{l}\right|,\varepsilon )}\right\|\right.}_{1}+{\left\|\left.\frac{\nabla L_{h}}{\max(\left|\nabla I_{h}\right|,\varepsilon )}\right\|\right.}_{1}$$

∇ in the formula represents the first order derivative operator in the horizontal and vertical directions, with a small constant ε introduced to ensure the validity of the function (typically set to 0.01 during the calculation). The term smoothness measures the relative structure of the illumination with respect to the input. As $L_{h}$ and $L_{l}$ differ due to the different intensities of illumination, but are structurally consistent with each other, the mutually consistent loss function is constructed as follows:

(3) $$\left\{\begin{array}{l} L_{mc}^{D}=\left\|{\left.M\cdot \exp(-c\cdot M)\right\|}_{1}\right.\\ M=\left|\nabla L_{l}\right|+\left|\nabla L_{h}\right| \end{array}\right.$$

The function f(x)=x·exp(−cx) used in the formula is controlled by the positive number c to control the shape of the function, and the overall trend is to first rise to the highest point and then fall to 0. The ideal value of c in this algorithm is 10 through experiments. In addition, the layer decomposition network needs to constrain the reconstruction error, i.e., the error before and after image decomposition should be small, so the loss function for reconstruction error is constructed as follows:

(4) $$L_{re}^{D}=\left\|{\left.I_{l}-R_{l}\cdot L_{l}\right\|}_{1}+\left\|{\left.I_{h}-R_{h}\cdot L_{h}\right\|}_{1}\right.\right.$$

Based on the above analysis, the loss function of the layer decomposition network is derived as:

(5) $$L^{D}=L_{re}^{D}+w_{rs}L_{rs}^{D}+w_{mc}L_{mc}^{D}+w_{is}L_{is}^{D}$$

The equations $w_{rs}$, $w_{is}$ and $w_{mc}$ are the weighting factors of the reflectance loss function, the light smoothing loss function and the mutual consistency loss function, respectively. The better results were obtained through several experiments with $w_{rs}$ = 0.009, $w_{is}$ = 0.2 and $w_{mc}$ = 0.15.

### 2.2. Light-Adjusted Network

As with the problems encountered in layer decomposition networks, there is no such thing as an optimal brightness as a reference standard for the light map. Therefore, to enable flexible adjustment of the light map, the intensity ratio α is introduced, which is formulated as follows:

(6) $$\alpha =\frac{L_{t}}{L_{s}}$$

$L_{s}$ is the original illumination map, $L_{t}$ is the target illumination map, and α is greater than 1 if you want to enhance the luminance based on the original illumination map, and conversely, α is less than or equal to 1. α is used as an indicator to train the adjustment function from the original illumination map to the target illumination map. The illumination adjustment network consists of three consecutive convolutional layers plus a ReLU activation layer, followed by a 1 × 1 convolutional layer to adjust the number of channels in the output, and finally, the illumination feature map is output after activation using the Sigmoid function. The loss function of the network is:

(7) $$L^{A}=MSE(\overset{\wedge }{L},L_{t})+MSE(\nabla \overset{\wedge }{L},\nabla L_{t})$$

The *MSE* (mean square error) in the formula is the mean square error function, $\overset{\wedge }{L}$ is the result of the network input of the low-light map $L_{l}$ adjusted by the network, and $L_{t}$ is the high-light map $L_{h}$.

### 2.3. Reflectivity Recovery Network

The loss function for the reflectance recovery network is first constructed. Since there is no so-called standard reflectance image, the reflectance image of a highly illuminated image decomposed by a layer decomposition network is used as a reference. The first term in the loss function is determined in terms of the similarity of the pixel values:

(8) $$L_{mse}^{R}=MSE(R_{h},\overset{\wedge }{R})$$

where $\overset{\wedge }{R}$ represents the output of the low-light reflectance image $R_{l}$ after processing by the reflectance recovery network. In addition to similarity in pixel values, the structure of the image after reflectance image recovery needs to be consistent, therefore, the second term of the loss function should be constructed as:

(9) $$\left\{\begin{array}{l} L_{dsim}^{R}=1-SSIM(R_{h},\overset{\wedge }{R})\\ SSIM(x,y)=\frac{\left(2\mu_{x}\mu_{y}+c_{1})(2\sigma_{xy}+c_{2}\right)}{\left(\mu_{x}^{2}+\mu_{y}^{2}+c_{1})(\sigma_{x}^{2}+\sigma_{y}^{2}+c_{2}\right)}\\ c_{1}={\left(k_{1}L\right)}^{2},c_{2}={\left(k_{2}L\right)}^{2} \end{array}\right.$$

where *SSIM* (structural similarity) is the structural similarity function.

$\mu_{x}$ and $\sigma_{x}^{2}$ are the mean and variance of variable x, $\mu_{y}$ and $\sigma_{y}^{2}$ are the mean and variance of variable y, and $\sigma_{xy}$ is the covariance of x and y. $c_{1}$ and $c_{2}$ are two constants used to prevent the denominator from being zero, where L is the range of pixel values, with $k_{1}$ taken as 0.01 and $k_{2}$ as 0.03. The closer the trained reflectance image is to the reference, the larger the value of *SSIM*, and the smaller the value of $L_{dsim}^{R}$, in line with the purpose of network training. The loss function of the reflectance recovery network can be obtained from the above analysis:

(10) $$L^{R}=L_{mse}^{R}+L_{dsim}^{R}$$

**Figure 2:**
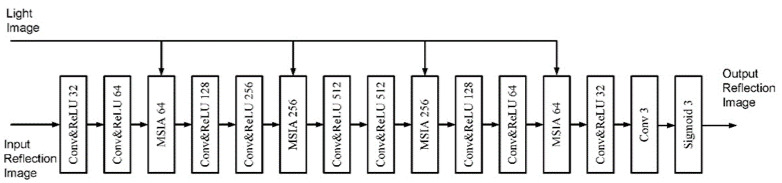
Reflectance restoration net.

The biggest difficulty for the reflectance recovery problem is that the degradation distribution of the reflectance image is complex. The high illumination part is less degraded, and the low illumination part is severely degraded. Therefore, in order to better recover the reflectance image, it is necessary to introduce the illumination information together with the degraded reflectance into the recovery network. The structure of the reflectance recovery network is illustrated in Figure 2.

**Figure 3:**
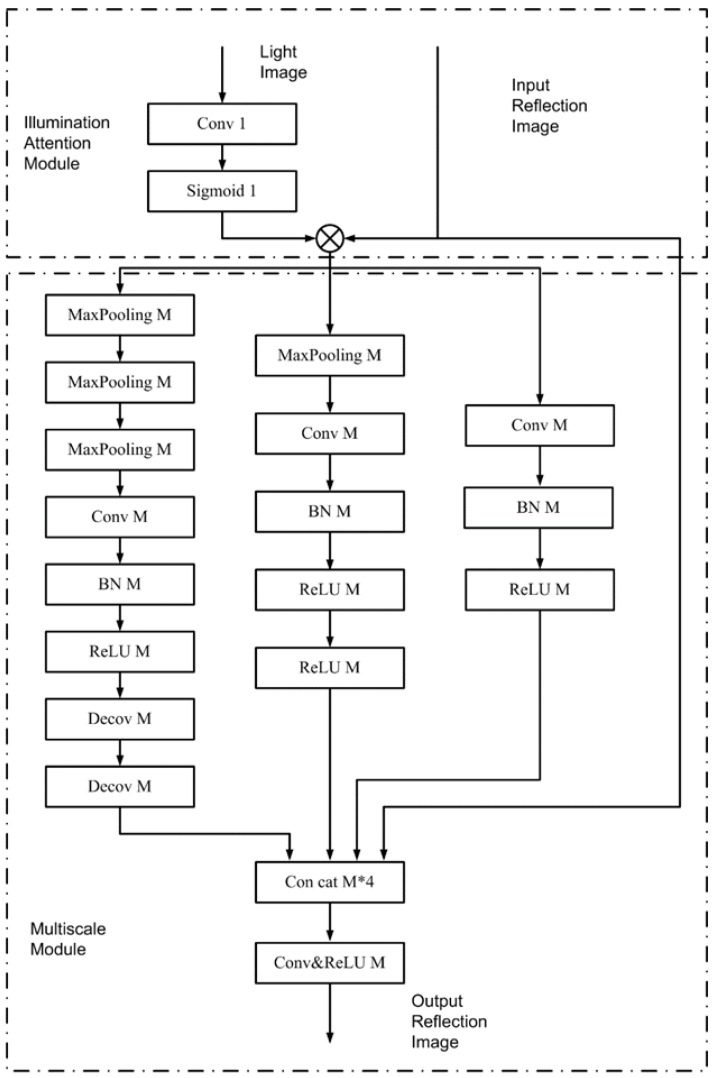
Multi-scale illumination attention module.

The network consists of 10 convolutional layers and 4 multi-scale illumination attention (MSIA) modules. The biggest improvement of the KinD++ network over the KinD network is the reflectance recovery network. In the KinD network, the reflectance recovery network is structured in a U-Net-like shape, enabling the noise reduction and color correction of reflectance images. However, for some images, the processed reflectance images can suffer from problems such as overexposure and halo artefacts. The MSIA module was therefore introduced into KinD++’s reflectance recovery network to ameliorate this deficiency, and the MSIA module is shown in Figure 3. The MSIA module consists of two sub-modules, the illumination attention module and the multi-scale module. The illumination attention module guides the network to deal with heavily degraded areas, while the multi-scale module is responsible for extracting richer features from the reflectance image to recover color and detail.

## 3. Data Augmentation and Anchor Box Optimization

### 3.1. Data Augmentation

**Figure 4:**
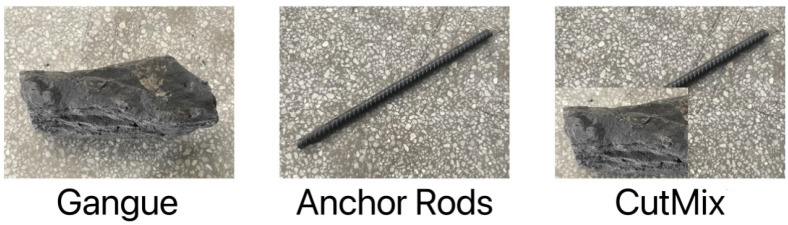
CutMix pictures.

YOLOv4 uses three kinds of data augmentation: CutMix [21], Mosaic and DropBlock [22] regularization. CutMix performs data augmentation from the perspective of image blending, through which the strategy makes the trained target detection network not overly dependent on certain features of the target, increases the detection capability of occluded targets, and improves the generalization and target localization of the trained model. CutMix obtained a blended image by cutting and pasting a gangue image block onto the anchor image to mask it, as shown in Figure 4. The area of the cut graph is determined by a preset percentage value, and the value of the label is determined by the proportion of the fused area of the current picture content. In Figure 4, 30% and 70% of the two images are fused together, and the original labels are [1,0] and [0,1] respectively, so the fused labels are [0.3,0.7].

**Figure 5:**
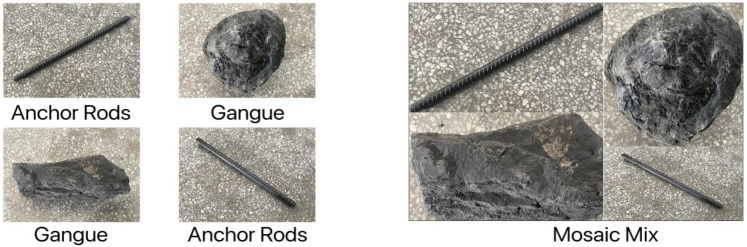
Mosaic mixture plot.

Similar to the CutMix method, Mosaic also performs dataset enhancement by blending the target images. However, unlike CutMix which blends two images, Mosaic does so by randomly cropping four images before stitching them onto a single image. This strategy enriches the background of the target object and enhances the detection of objects that reveal only some of their features, indirectly improving the batch value at training time, as shown in Figure 5. Since the main idea of this method is to randomly crop four pictures and then splice them into one picture as training data, the specific size cannot be given.

Unlike the two aforementioned data augmentation approaches that perform blending on the initial image, DropBlock regularization performs the random discarding of block features from the feature map level during training, enhancing the robustness and generalization of the training model.

### 3.2. Anchor Box Optimization

The YOLOv4 target detection framework is the final solution based on YOLOv3 by adding various tuning tools. YOLOv4 retains the Darknet53 framework structure of YOLOv3 in the network backbone but introduces the cross stage partial network (CSPDarknet53), which improves the backbone structure to CSPDarknet53, which reduces the computational effort and improves the detection accuracy. The next section describes the principles of YOLOv4 in terms of data augmentation, network structure, bounding box regression function and loss function, respectively.

The YOLOv4 target detection algorithm provides nine sets of anchored rectangular box width and height values. For the dataset of large gangue and anchor rods on the belt conveyor collected in this paper, the anchor box dimensions of the COCO dataset provided by YOLOv4 cannot be used directly, and the dataset with the bounding boxes calibrated needs to be clustered to obtain the anchor box dimensions of the anchor rods and gangue in this paper for subsequent network training.

The K-means algorithm is a classical unsupervised clustering algorithm that is able to group similar objects into the same cluster. The K-means algorithm is generally used to process vector data, using Euclidean distance as a metric, but the aim of this paper is to obtain the width and height values of the anchor boxes. Therefore, the intersection ratio of the width and height of the bounding box (IOU) is used as the metric.

**Figure 6:**
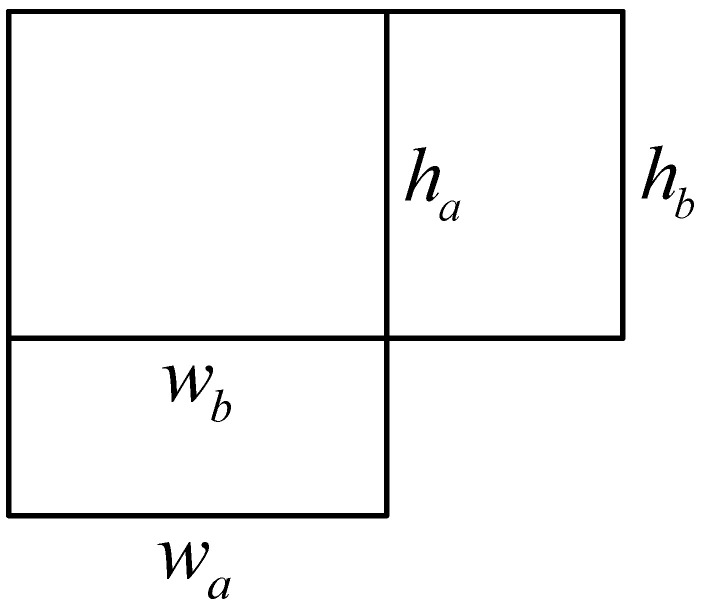
The measurement method of the rectangular box in the clustering algorithm.

As shown in Figure 6, Equation (11) is the formula for calculating the intersection and ratio of two bounding boxes with both upper left corners at the origin, which is substituted into Equation (12), and this distance variable distance is used in place of the Euclidean distance in the K-means algorithm.

(11) $$\begin{array}{c} IOU(a,b)=\frac{intersection(a,b)}{union(a,b)-intersection(a,b)}\\ \;\;\;\;\;\;=\frac{\min(w_{a},w_{b})\cdot \min(h_{a},h_{b})}{w_{a}\cdot h_{a}+w_{b}\cdot h_{b}-\min(w_{a},w_{b})\cdot \min(h_{a},h_{b})} \end{array}$$

(12) distance(a,b)=1−IOU(a,b)

K in K-means denotes the number of different clusters to be found, which is set manually according to the requirements, and is performed as follows:

Step 1: randomly select K anchor box widths and heights from the labeled dataset as the starting clustering centers, with the number of anchor box width–height pairs as $C=\{c_{1},c_{2},\cdots ,c_{k}\}$, where $c_{i}=(w_{i},h_{i})$.

Step 2: for each sample $x_{i}=(w_{i},h_{i})$ in the foreign matter dataset, calculate the distance distance from it to the K clustering centers and assign it to the class with the smallest distance.

Step 3: for each class $c_{1}$, recalculate the mean value of the width–height of the anchor boxes in this class as the width–height of the anchor boxes in the new clustering center.

Step 4: repeat Steps 2 and 3 until the width and height of the central anchor box does not change.

From the above process, we can see that the problem with K-means is that the width and height of the initial clustering center anchor box needs to be selected artificially, and different initial centers may bring different clustering results. To address this problem, this paper uses the K-means++ algorithm to improve it. The specific execution steps of K-means++ are as follows:

Step 1: the width and height of a randomly selected bounding box from the annotated dataset is used as the cluster center, and the anchor box is denoted as $c_{1}$.

Step 2: calculate the minimum distance between each bounding box and the currently existing cluster center bounding box (i.e., distance of the cluster center anchor box with the closest value of width and height), here denoted as D(x). Next, calculate the probability of each bounding box being selected as the next cluster center anchor box as Equation (13), χ for the whole dataset. Finally, the width and height of the next cluster center anchor box is selected by the roulette wheel method.

Step 3: repeat the second step until K initial cluster center anchor box width and height values are selected.

Step 4: perform Steps 2 to 4 in the K-means algorithm.

(13) $$\frac{D{\left(x\right)}^{2}}{\sum_{x\in \chi }D{\left(x\right)}^{2}}$$

## 4. Experiments and Analysis

### 4.1. KinD++ Algorithm Experimentation and Analysis

#### 4.1.1. Dataset Production

For the field of low-light image enhancement, a common public dataset is the LoL (low-light) dataset.

**Figure 7:**
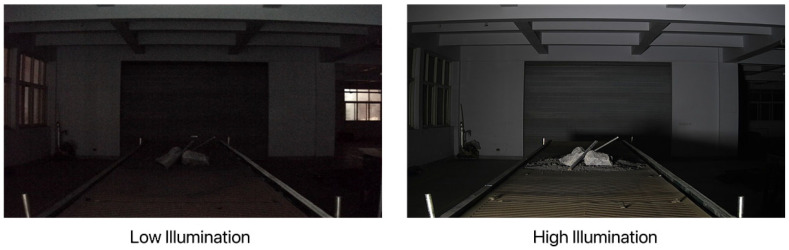
Different lighting images collected by the test bench.

**Figure 8:**
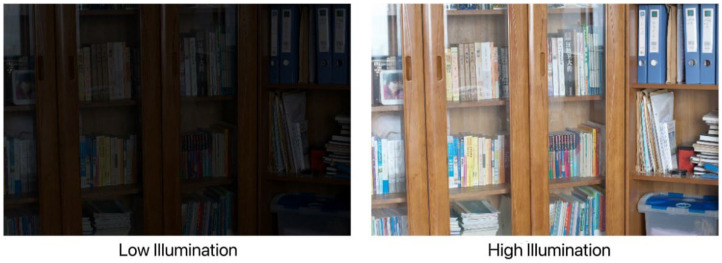
Different lighting images in the LOL dataset.

In this experiment, 400 low/normal light image pairs were obtained by controlling the light intensity and angle on the belt conveyor foreign object detection and localization test bench, which combined with 500 pairs in the LoL dataset resulted in a dataset of 900 low–high-light image pairs. Some images of the produced dataset are shown in Figure 7 and Figure 8:

#### 4.1.2. Training Setup

As there are three sub-networks in the KinD++ network, and the training is performed in steps. The layer decomposition network is trained first, followed by the illumination adjustment network and finally the reflectance recovery network. The gradient descent method was optimized using the Adam optimizer when back-propagating during training. The training parameters for the three networks are shown in Table 1. Patch-size denotes the basic unit of image processing by the network. The batch-size indicates the number of images processed at each parameter update, and the Epoch value indicates the number of traversals of the training set. LR (learning rate) is the learning rate and is set to 0.0001.

#### 4.1.3. Results and Analysis

In order to verify the enhancement effect of the KinD++ algorithm for low-light images, the KinD model and the Retinex-Net model were trained using the same training set as KinD++. In this experiment, low-light images were collected for enhancement in four environments: dark with auxiliary light, evening without light, evening with light and daytime backlight.

**Figure 9:**
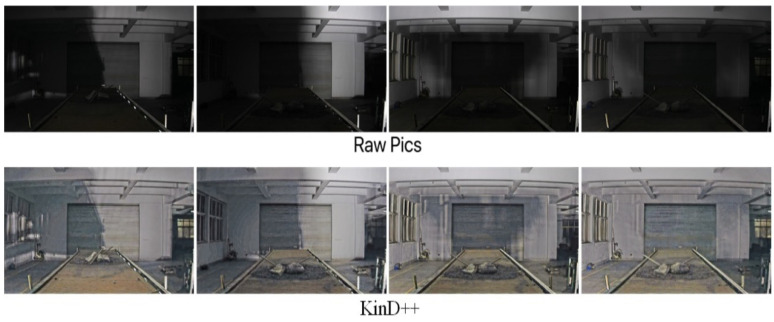
Enhancement of KinD++ algorithm on images in the dark.

The results of the low-light image enhancement in a dark environment with an auxiliary light source are shown in Figure 9. After processing by the KinD++ algorithm, the image can recover the original appearance very well, and the details of foreign objects such as anchor rods and large gangue on the belt conveyor are particularly well recovered.

**Figure 10:**
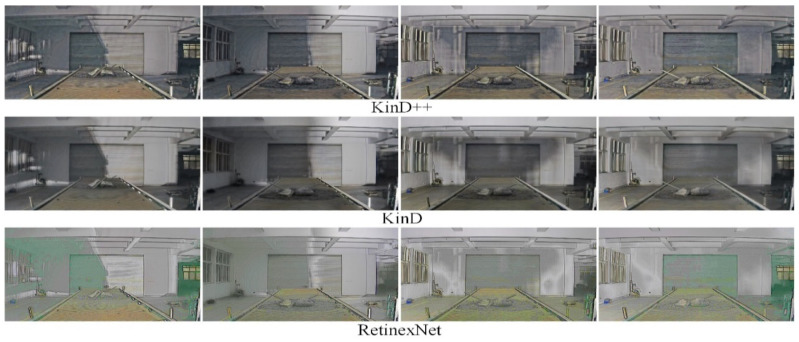
Enhancement of images in the dark by deep learning algorithms.

**Figure 11:**
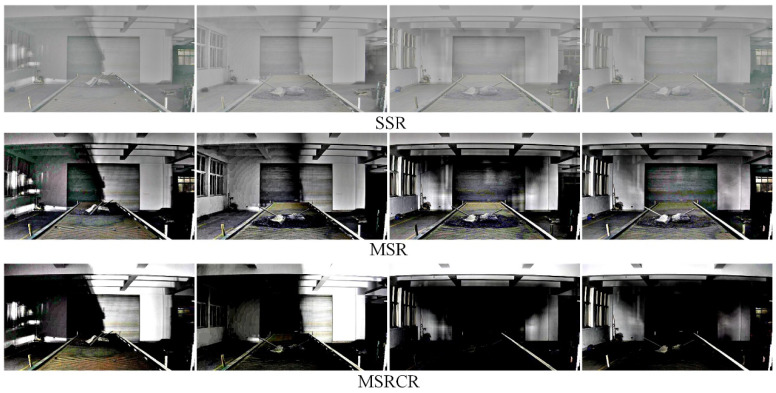
Enhancement of images in the dark by traditional algorithms.

Figure 10 shows a comparison of the enhancement effect of the three neural network-based deep learning algorithms on the original image in Figure 9. It can be seen from Figure 10 that the KinD++ algorithm and the KinD algorithm are significantly more effective than the Retinex-Net algorithm and are able to recover more detail in the dark areas of the image. Figure 11 shows the results of three traditional low-light image enhancement algorithms based on Retinex theory. Comparing the results plotted in Figure 10 based on the convolutional neural network algorithm, the processing in Figure 11 is significantly less effective.

**Figure 12:**
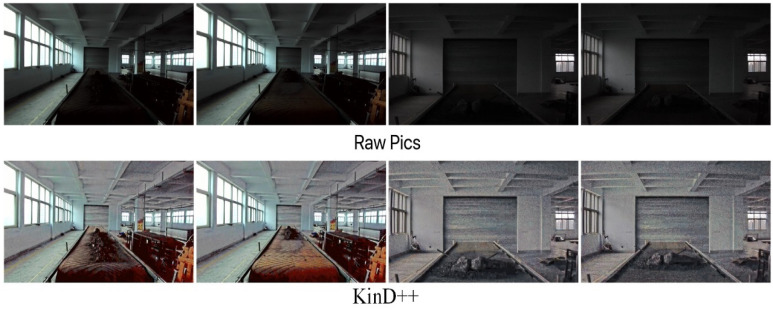
KinD++ algorithm for enhancement of evening without light source image.

**Figure 13:**
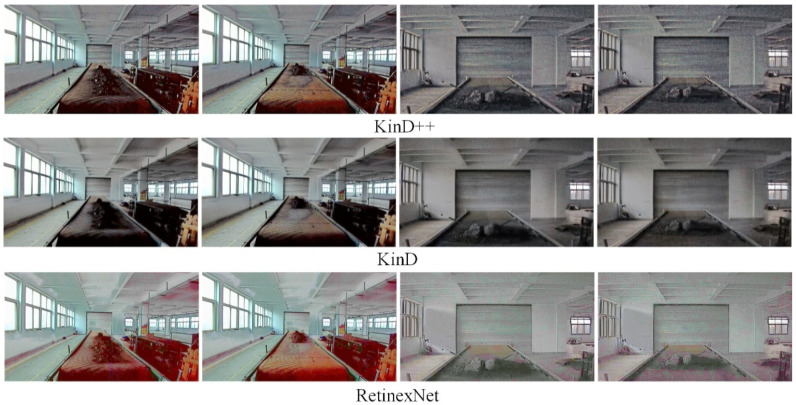
Deep learning algorithm for enhancement of evening images without light source.

The results of the low-light image enhancement in the evening without light are shown in Figure 12. As can be seen in Figure 12, the KinD++ algorithm is able to enhance the low-light image well in this environment, while the recovered image colors are also more accurate, and foreign objects such as anchors and gangue on the belt conveyor can be clearly observed after processing. As can be seen from Figure 13, the KinD algorithm is as effective as the KinD++ algorithm in recovering the details of foreign objects.

**Figure 14:**
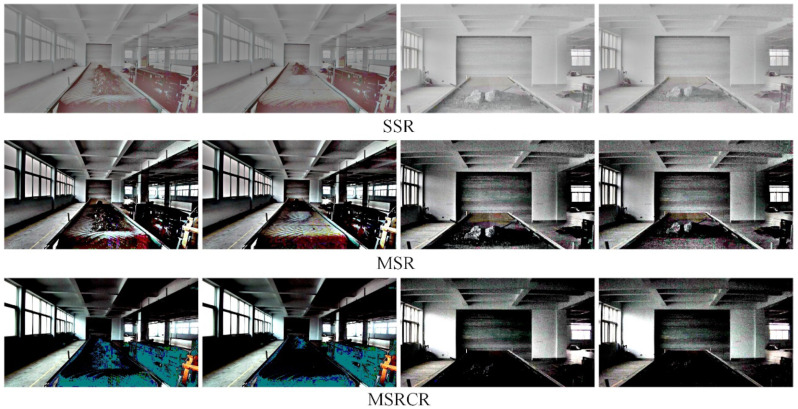
Enhancement of traditional algorithms for evening images without light source.

Figure 14 shows a comparison of the enhancement effect of three conventional algorithms based on Retinex theory for the original image in Figure 12. It can be seen from the figure that the enhancement effect of these three algorithms is much less than the enhancement effect of the neural network-based algorithm.

**Figure 15:**
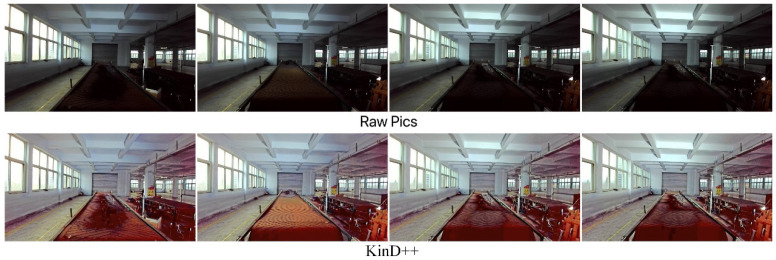
Enhancement of images with light sources in the evening by KinD++ algorithm.

**Figure 16:**
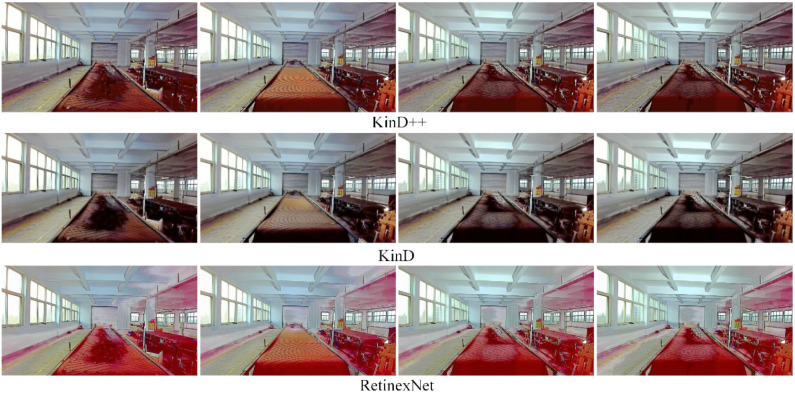
Enhancement of images with light sources in the evening by deep learning algorithm.

The results of the low-light image enhancement in an environment with an auxiliary light source in the evening are shown in Figure 15. It can be seen from the figure that the KinD++ algorithm is able to enhance the low-light image better. Figure 16 shows the enhancement of the original image in Figure 15 by the three convolutional neural network-based algorithms, and it can be seen that the colors of the KinD++ enhanced image are closest to the original colors. The Retinex-Net algorithm, on the other hand, has more artefacts and noise in the enhanced image.

**Figure 17:**
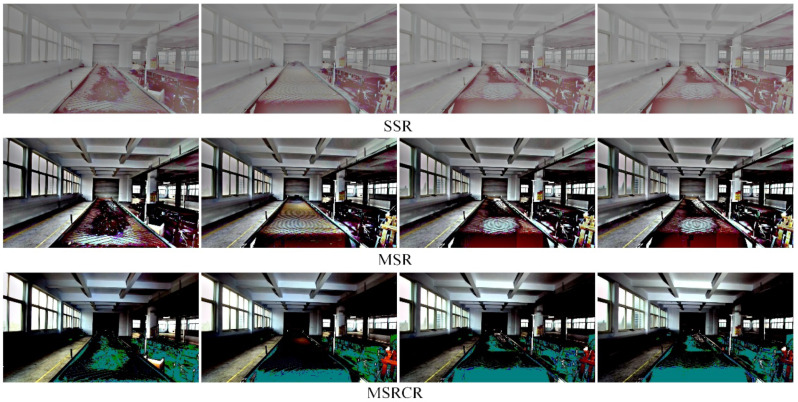
Enhancement of images with light sources in the evening by traditional algorithms.

Figure 17 shows a comparison of the enhancement effect of three conventional algorithms based on Retinex theory on the original image in Figure 15. Compared with Figure 16, the enhancement effect of the traditional algorithms is inferior to that of the convolutional neural network-based algorithms. The color of the SSR processed image is almost completely lost, with a greyscale image, there are blurred details of the object and serious loss of features. The color distortion is severe.

**Figure 18:**
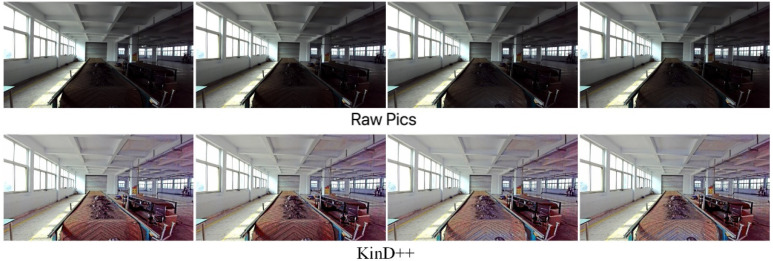
Enhancement of KinD++ algorithm on daytime backlight image.

**Figure 19:**
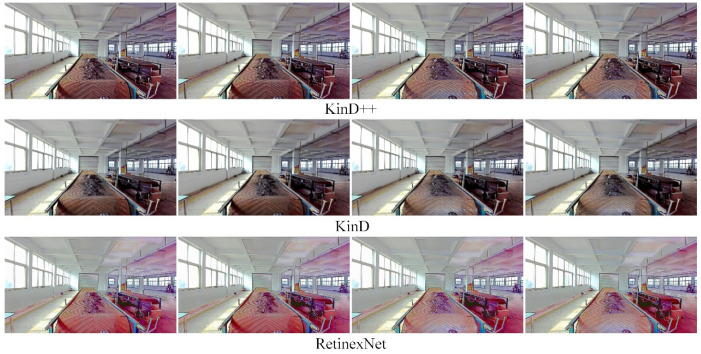
Enhancement of daytime backlit images by deep learning algorithms.

The results of the low-light image enhancement in a daytime backlit environment are shown in Figure 18. KinD++ was able to successfully enhance the daytime backlighting image, and the features of objects such as gangue and coal on the belt conveyor that were in darkness were well recovered, as can be seen from the enhancement effect graph in Figure 18. Figure 19 shows the enhancement effect of the deep learning algorithm on the original image. From the comparison results, it can be seen that the KinD++ algorithm and the KinD algorithm enhancements are significantly better than those of the Retinex-Net algorithm.

**Figure 20:**
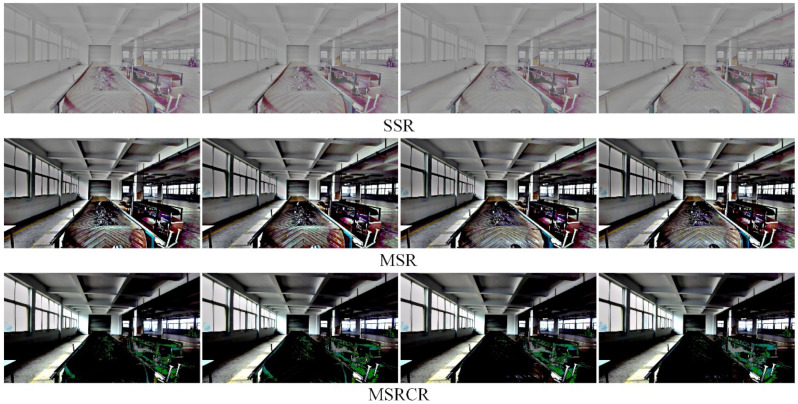
Enhancement of daytime backlight images by traditional algorithms.

Based on Retinex theory, Figure 20 compares the enhancing effects of three common algorithms on the original image. Compared with Figure 19, the traditional low-light image enhancement algorithm has an inferior enhancement effect on daytime backlit environment images than the deep learning algorithm. The SSR algorithm enhances the dark areas while losing a large amount of color, and the object features are not well recovered and are very blurred. The MSR algorithm recovered some color, but the color distortion was severe, and the dark areas were poorly recovered, with more object features lost. MSRCR had the worst enhancement effect, with distorted color recovery and similarly no enhancement in dark areas.

The enhancement effect of the above four low-light environments was combined and the six algorithms were compared. It can be seen that the low-light image is enhanced by the KinD++ network, and that the dark features in the image are restored in detail. The brighter areas of the original image do not appear overexposed either, demonstrating the KinD++ network’s idea of zonal enhancement. In addition, the larger and more uniform the range of illumination, the better the enhancement effect of the KinD++ algorithm, which suggests that we should even out the light source and try to increase the range of illumination.

### 4.2. Target Detection Experiments and Analysis

#### 4.2.1. Dataset Extension Enhancement

**Figure 21:**
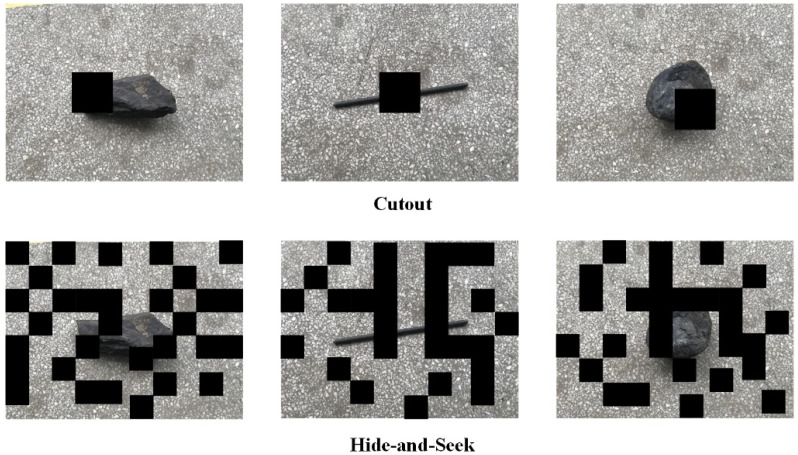
Image random erase processing.

While the majority of the huge gangue and anchor rods in the coal belt conveyor may be partially or completely covered throughout the coal transportation operation, they mostly show up in their entire shape in the photographs that were taken. Therefore, the foreign objects in the captured images are masked to simulate their appearance in real working conditions to enhance the training model’s ability to recognize semi-buried foreign objects. It also increases the number and type of training sets, effectively preventing the model from learning only some of the salient features of the foreign objects and improving the generalization capability of the model. The main methods to simulate object occlusion for data augmentation are random erasure [19], cutout [20], and hide-and-seek [23]. The main reason for the invalid data generated by both cutout and hide-and-seek methods is the random nature of their occlusion block positions. There is no guarantee that a valid occlusion image will be produced consistently, and they are unstructured occlusion operations. In order to implement data augmentation methods for masking while avoiding the problems of invalid masking in cutout and hide-and-seek methods, a structured masking strategy with grid masks is used. Figure 21 shows the schematic diagram of Image random erase processing.

**Figure 22:**
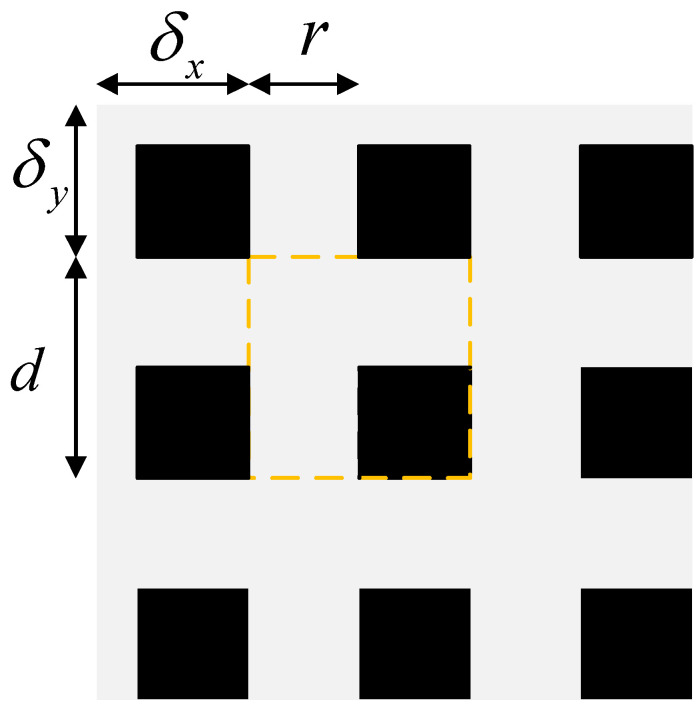
Schematic diagram of GridMask.

In order to implement a data augmentation approach to masking while avoiding the problems of invalid masking that occur in the cutout and hide-and-seek approaches, a structured masking strategy of a grid mask is used. As shown in Figure 22, the basic cell of the grid mask is the structure shown in the orange dashed box, and this basic cell is tiled to form the complete mask. The grey pixel value in the figure is 1 and the black pixel value is 0. The parameters r, d, $\delta_{x}$ and $\delta_{y}$ define the size and position of the first complete basic cell in the grid mask. r represents the ratio of the short grey edge in the basic cell to the edge length d, which determines the retention ratio k of the image. The retention ratio is defined as the ratio of the sum of the pixels retained in the image to the sum of the pixels in the whole image. The formula is 14, with M indicating the number of grey pixels retained in the mask, and H and W indicating the height and width of the image. When considering a grid mask consisting of an integer number of basic cells, the retention ratio k is related to r as shown in Equation (15).

(14) $$k=\frac{sum(M)}{H\times W}$$

Thus r determines the retention ratio after the image has been masked. Too large a retention ratio of the grid mask will result in targets in the image being unaffected by the masked blocks and will not avoid the overfitting problem of the neural network. In contrast, too small a retention ratio with too large a block will lead to the introduction of invalid data into the training dataset and the model will not converge during training, so a suitable R1 needs to be determined experimentally. The retention ratio is generally fixed during the training process, so the value of r is a constant.

(15) $$k=1-{\left(1-r\right)}^{2}=2r-r^{2}$$

The parameter d is the edge length of the basic cell and the magnitude of its value does not affect the retention ratio, so a dynamic value can be used, as shown in Equation (16) to set a range of minimum and maximum and randomly determine an d value within the range when the masked image expands the dataset.

(16) $$d=random(d_{\min},d_{\max})$$

The parameters $\delta_{x}$ and $\delta_{y}$ indicate the distance from the top left corner of the first complete basic cell to the edge of the image. In order to make the masking of the grid mask more likely, these two distance parameters are also taken randomly and limited in range. The ranges are shown in Equation (17).

(17) $$\delta_{x}(\delta_{y})=random(0,d-1)$$

Once the values of r, d, $\delta_{x}$ and $\delta_{y}$ have been determined, the size, retention ratio and position of the basic cells are also determined, and the grid mask for tiling the basic cells is also determined. When the mask is determined and multiplied by the original image, the image is obtained after the grid mask is masked.

(18) $$\tilde{x}=x\times M$$

**Figure 23:**
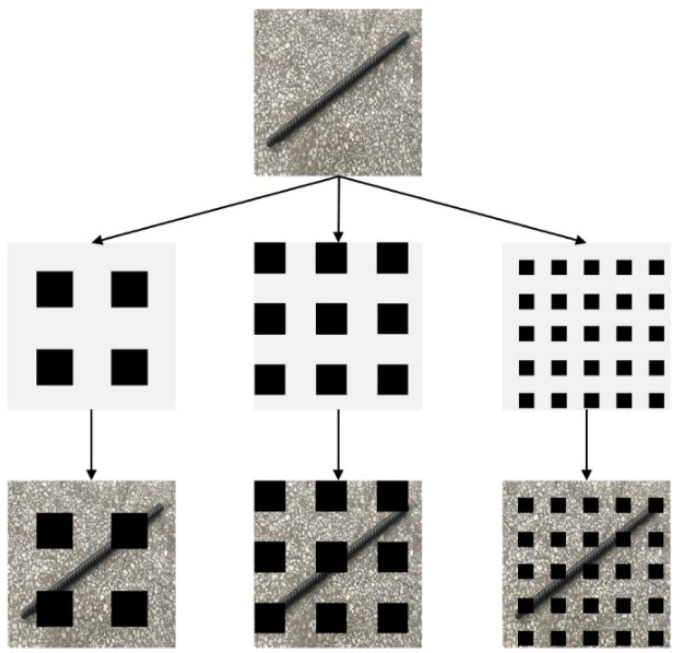
Multiply the image with the GridMask.

As in Equation (18), x is the original image, M is the grid mask and the range of values is M∈(0,1), i.e., the grey block has a pixel value of 1 and the black block has a pixel value of 0. The result of multiplying the image with the mask is shown in Figure 23, with the three different grid masks controlled by the size of the parameters.

#### 4.2.2. Experiments and Analysis of Results

**Figure 24:**
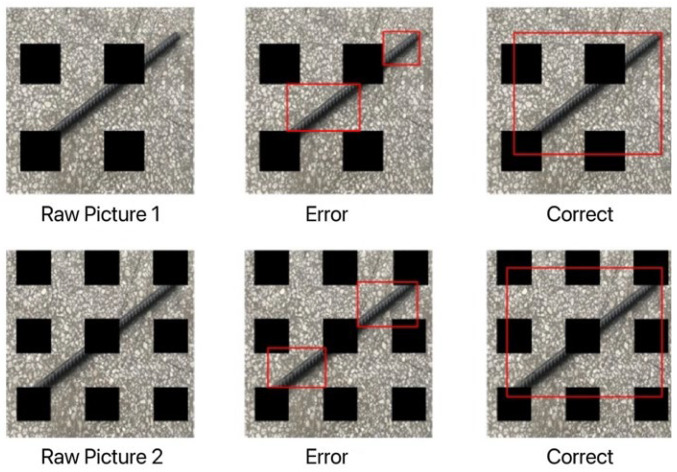
Bounding box annotations for occluded images.

This paper collects 4000 pictures of foreign objects contained on belt conveyors. Of these, 1500 images contain only anchor rods, 1500 images contain only gangue, and 1000 images contain both anchor rods and gangue. A total of 1000 images were extracted from each of the anchor-only and gangue-only images and masked using the GridMask data augmentation method. The labelImg software was used to annotate the image dataset to obtain 6000 labels written in XML language, and these labels were randomly disordered and divided into the training set, validation set and test set, accounting for 60%, 20% and 20% respectively. Figure 24 shows that when the image is masked by the grid, the labeled bounding box is the original size of the target, and not two bounding boxes because the masking makes the target split into two parts.

For the anchor box optimization experiment, the 6000 XML language written labels obtained above using labelImg software were fed into the K-means++ algorithm to obtain nine anchor boxes, the results of which are shown in Table 2.

**Figure 25:**
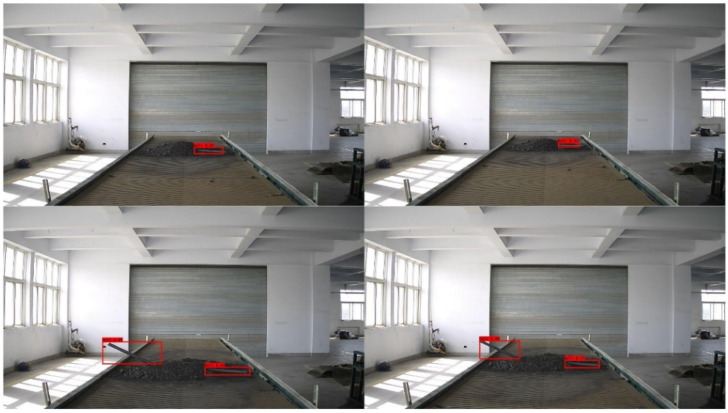
Semi-buried bolt detection.

**Figure 26:**
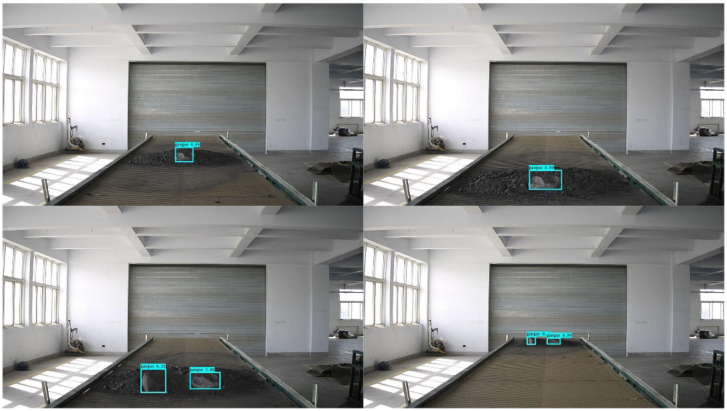
Semi-buried gangue detection.

**Figure 27:**
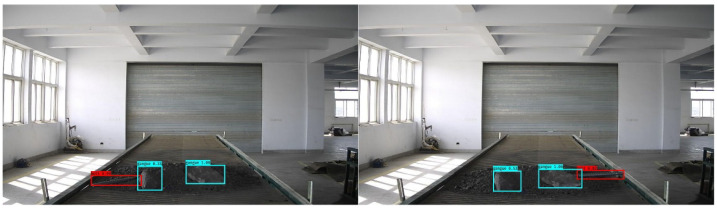
Semi-buried bolt and gangue detection.

Figure 25, Figure 26 and Figure 27 show the results of the YOLOv4 model for the detection of foreign objects on a belt conveyor, from which it can be seen that the improved model is able to detect single and multiple foreign objects with high accuracy.

## 5. Conclusions

In this study, the KinD++ network was used to implement image enhancement for low-light scenes. In the experimental part, multiple algorithms were used to enhance images in different lighting environments. A comparison of the results shows that the deep learning algorithms based on convolutional neural networks are much more effective in enhancing low-light images than traditional algorithms. The comparison of the three deep learning algorithms shows that the excellent overall performance of the KinD++ network in low-light image enhancement is able to recover image details in the dark well, providing a distinctly characterized image dataset for subsequent target detection. The study then optimizes the anchor boxes by the K-means++ algorithm. It experimentally determines the width and height values of the nine anchor boxes and the parameter values of the grid mask for the dataset of this paper, obtaining better optimization results and enhancing the generalization capability and accuracy of the model. Finally, the anchor boxes YOLOv4 algorithm was used to detect large gangue and anchor rods on the belt conveyor under various scenarios, and better results were obtained.

## Figures and Tables

**Table 1:** KinD++ network training parameter setting.

	Patch-Size	Batch-Size	Epoch	LR
Layer decomposition network	48	10	2000	0.0001
Illumination adjustment network	48	10	2000	0.0001
Reflectance recovery network	384	4	1000	0.0001

**Table 2:** Dataset anchor box width and height values.

	Small Target	Medium Target	Big Target
Original anchor box	10,13;16,30;33,23	30,61;62,45;59,119	116,90;156,198;373,326
This article anchor box	18,28;32,37;33,13	39,18;50,22;61,31	73,44;88,10;116,54

## Data Availability

The data that support the findings of this study are available from the corresponding author upon reasonable request.
